# Severe hyperammonemia from intense skeletal muscle activity: A rare case report and literature review

**DOI:** 10.1097/MD.0000000000017981

**Published:** 2019-11-22

**Authors:** Vikas Taneja, Haneesh Jasuja

**Affiliations:** a Orlando Regional Medical Center, Orlando, FL; b Materials and Nanotechnology Program, North Dakota State University, Fargo, ND.

**Keywords:** exercise, hyperammonemia, skeletal muscle

## Abstract

**Rationale:**

Adult hyperammonemia is most often the result of hepatic dysfunction. Hyperammonemia in the setting of normal hepatic function is a much less common phenomenon and has usually been associated with medications and certain disease states. Here, we present an unusual case of severe hyperammonemia caused physiologically by intense muscle activity in a patient lacking any evidence of liver disease.

**Patient concerns:**

A 36-year-old woman was brought to the emergency department for a suicide attempt after being found covered in Lysol and Clorox germicidal bleach. She was noted to be in a state of violent psychosis with extreme agitation and had to be sedated and intubated for airway protection.

**Diagnosis and interventions:**

Initial labs revealed hyperammonemia, lactic acidosis, and anion gap metabolic acidosis. Aminotransferases, bilirubin, and creatine kinase (CK) were normal. Renal function, prothrombin time, activated partial thromboplastin time, and international normalized ratio were also unremarkable and remained so at 24 hours. Ethyl alcohol, acetaminophen, salicylate, and valproic acid were all undetectable in blood. She received 2 doses of lactulose overnight, with a subsequent bowel movement. Next day, her mentation, serum ammonia level, and lactic acid level were back to normal, and she was extubated. Aminotransferases and CK levels were elevated but improved with supportive care. A detailed history and relevant biochemical investigations were unremarkable for any other etiology of hyperammonemia including the common inborn errors of metabolism (IEM). The combination of clinical findings of extreme skeletal muscle activity along with hyperammonemia and lactic acidosis, and subsequently rhabdomyolysis in the setting of unremarkable history and otherwise normal hepatic function strongly suggest the myokinetic origin of hyperammonemia in the patient.

**Outcome:**

The patient recovered well with supportive care and was discharged on day 5.

**Lessons:**

This unique case illustrates the important role of skeletal muscle in the human metabolism of ammonia. In our discussion, we also elucidate the underlying pathophysiology, with the objective of improving clinician understanding of various differential diagnoses.

## 1 Introduction

Hyperammonemia has been well documented to be involved in the etiology of hepatic encephalopathy, sometimes heralding transition from liver injury to hepatic failure. The liver is a vital organ involved in ammonia metabolism. Ammonia is primarily generated in the gastrointestinal (GI) tract with a minor contribution from renal tubules and is eventually metabolized into urea by hepatocytes before being renally excreted. This coordination is responsible for maintaining a state of ammonia homeostasis in the body. In adults, the most common etiology of disturbance of this state of equilibrium is hepatic dysfunction, impairing the body's ability to metabolize physiologically produced ammonia. Skeletal muscle usually consumes ammonia,^[1]^ but with activity it may itself become a producer causing clinically significant hyperammonemia.^[2]^ We present this case to highlight such a scenario with the aim of improving understanding of underlying pathophysiology and etiology of nonhepatic hyperammonemia.

## 2 Case

A 36-year-old woman was brought to the emergency department (ED) after a suicide attempt with Lysol daily cleanser (sodium chloride − <0.5%, hypochlorous acid − <0.05%) and Clorox germicidal bleach (sodium hypochlorite − 6.15%, sodium hydroxide − <1%). The patient was found in her room covered in these household chemicals along with partially empty bottles. She was extremely agitated and violent, and had to be restrained. En route to the hospital, she also received ketamine for sedation. In the ED, the patient was still agitated, but otherwise hemodynamically stable; vitals were noted to be: blood pressure − 162/94 mm Hg, heart rate − 108/minute, respiratory rate − 28/minute, and pulse oximetry − 90% on room air. She was subsequently sedated and intubated for airway protection. Laboratory studies revealed hyperammonemia and anion gap metabolic acidosis (arterial pH − 7.11 on arterial blood gas analysis) secondary to lactic acidosis. Rest of the workup was unremarkable and is as follows (Table 1). Urine drug screen was positive for benzodiazepines and cocaine. Acute hepatitis profile was negative.

**Table 1:**
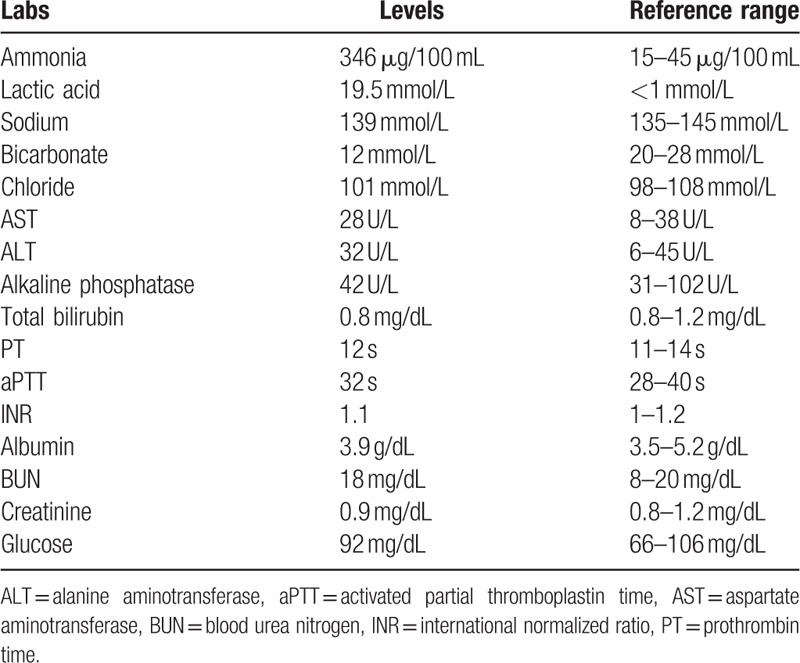
Initial laboratory values with reference range.

**Figure 1:**
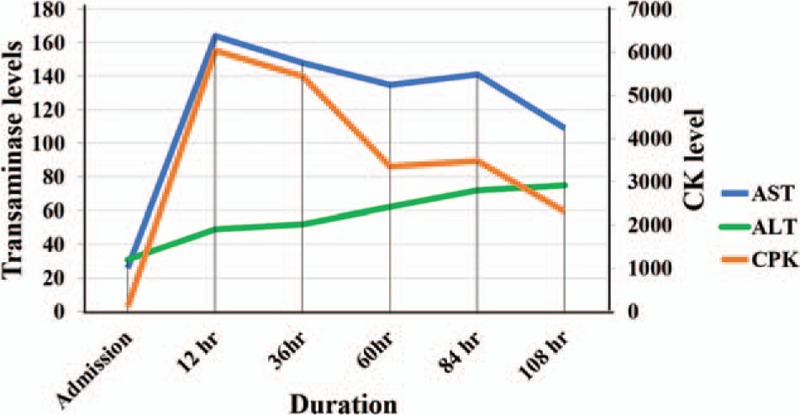
Graphical representation of temporal serum creatine kinase (CK) and aminotransferase trends during admission.

She subsequently underwent imaging including computed tomography (CT) of head, chest, and abdomen and pelvis, which did not show any abnormalities. Due to a concern of chemical ingestion, the patient promptly underwent urgent esophagogastroduodenoscopy (EGD), which did not reveal any evidence of caustic mucosal injury to suggest toxic ingestion. The patient received 2 doses of lactulose overnight through nasogastric tube and was noted to have a nonmelanotic bowel movement. Next morning, her mentation improved significantly, and she was extubated. Surprisingly, repeat ammonia level 12 hours postadmission was normal (lactic acid had also returned to the normal level at 3 hours). Aspartate aminotransferase (AST) level was noted to be elevated at 164 U/L, and creatine kinase spiked to 6039 U/L. She was continued on aggressive IV hydration with improvement in creatine kinase (5450 U/L) and AST (148 U/L) levels at 36 hours (Fig. 1). Repeat ammonia level was again unremarkable. The patient was complaining of vague right upper quadrant pain on day 3 and subsequently underwent a hepatobiliary iminodiacetic acid scan, which did not reveal any evidence of cholecystitis, cholelithiasis, or biliary dyskinesia. Fasting quantitative plasma amino acid analysis performed on day 3 revealed normal levels of citrulline = 30 μmol/L (reference range 16–51 μmol/L), arginine = 58 μmol/L (reference range 43–407 μmol/L), and glutamine = 510 μmol/L (reference range 428–747 μmol/L).

A detailed history was obtained later when the patient was back to baseline. She apparently drenched those disinfectants on herself to seek family's attention and did not actually ingest them. Social history was significant for rare alcohol intake, with her last drink being 3 months ago. She also denied smoking but had been using cocaine off and on for a few years. Medication review revealed infrequent acetaminophen intake and absence of any prescription medications in the preceding few weeks. She also denied any history of chronic abdominal pain, diarrhea, nausea/vomiting, or any specific food intolerances or any GI symptoms during early mornings, or after any fasting or after a protein-rich meal. She also denied any family history of liver disease. The patient reported that she had otherwise been a healthy child while growing up with appropriate height and weight (163 cm and 77.2 kg currently with a body mass index [BMI] of 29.06). Given the unremarkable work-up, hyperammonemia was most likely secondary to intense muscle activity during the psychotic episode. She was subsequently discharged to inpatient behavioral health unit on day 5 in stable condition.

## 3 Discussion

**Figure 2:**
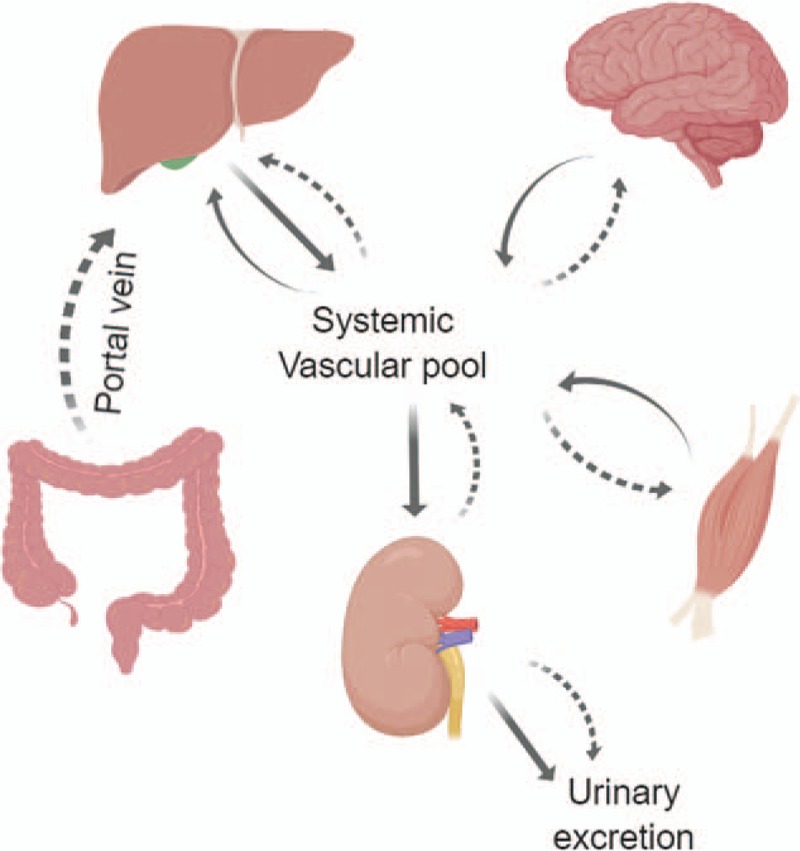
Schematic representation of interorgan transfer of ammonia and its metabolites in humans. Solid straight lines represent urea exchange, solid curved lines represent glutamine exchange, and dotted curved lines represent ammonia exchange.

Most of the ammonia in the human body is generated in the gut by bacterial metabolism and dietary protein digestion.^[2]^ Enterocytes, predominantly in small bowel, also produce ammonia from circulating glutamine.^[3]^ Small amounts are also produced by kidneys primarily in the proximal tubule, 60% to 70% in the normal basal state, and increasing to up to 80% in metabolic acidosis.^[4]^ Glutamine is again the primary substrate resulting in the generation of equimolar amounts of ammonium and bicarbonate ions.^[5]^ This ammonia is either excreted in urine or released in systemic circulation. Metabolic acidosis stimulates renal ammoniagenesis with proportionate urinary excretion where it combines with H^+^ ions to promote acid excretion.^[6]^ Ammonia is eventually taken up from the portal and systemic venous circulation by hepatocytes and incorporated into urea cycle to produce urea, which is excreted by kidneys.^[7]^ The brain also metabolizes small amounts of circulating ammonia, which crosses the blood–brain barrier utilizing glutamine synthetase (GS), which is exclusive to glia in central nervous system. Cerebral edema noted in hyperammonemia is largely the result of osmotic astrocyte swelling secondary to intracellular accumulation of glutamine. Interorgan exchange of ammonia exchange is shown in Figure 2.

## 4 Role of skeletal muscle in ammonia metabolism

Skeletal muscle is normally a net consumer of ammonia^[8]^ utilizing GS to produce glutamine. But with activity, it also starts producing ammonia. And when local GS activity is overcome, it becomes a net producer, as has been shown by the proportionate increase in ammonia levels with increasing exercise intensity.^[9]^ The etiology of this ammonia production is branched-chain amino acid catabolism^[10]^ and deamination of adenosine monophosphate (AMP).

Intensely exercising muscle can generate adenosine triphosphate (ATP) almost immediately using the adenylate kinase/myokinase reaction, which catalyzes the conversion of 2 adenosine diphosphate (ADP) molecules into 1 molecule of ATP and 1 molecule of AMP. If the muscle continues exercising, AMP is deaminated to inosine monophosphate (IMP) to maintain equilibrium for the upstream dephosphorylation reactions. The reaction is catalyzed by myoadenylate deaminase/AMP deaminase, with concurrent production of ammonia (NH_3_).^[11]^

2ADP → ATP + AMP
AMP + H_2_O → IMP + NH_3_

This ammonia is then released in the venous circulation either unchanged or combined with glutamate to produce glutamine, which is an energy-consuming step, and when muscle ATP is depleted, the proportion of ammonia release increases. Interestingly, this excess glutamine is in turn used by proximal tubule for ammoniagenesis, which may be used to counterbalance any metabolic acidosis from anaerobic skeletal muscle activity.^[12]^

## 5 Common differential diagnoses for hyperammonemia in adults

### 5.1 Hepatic dysfunction

**Figure 3:**
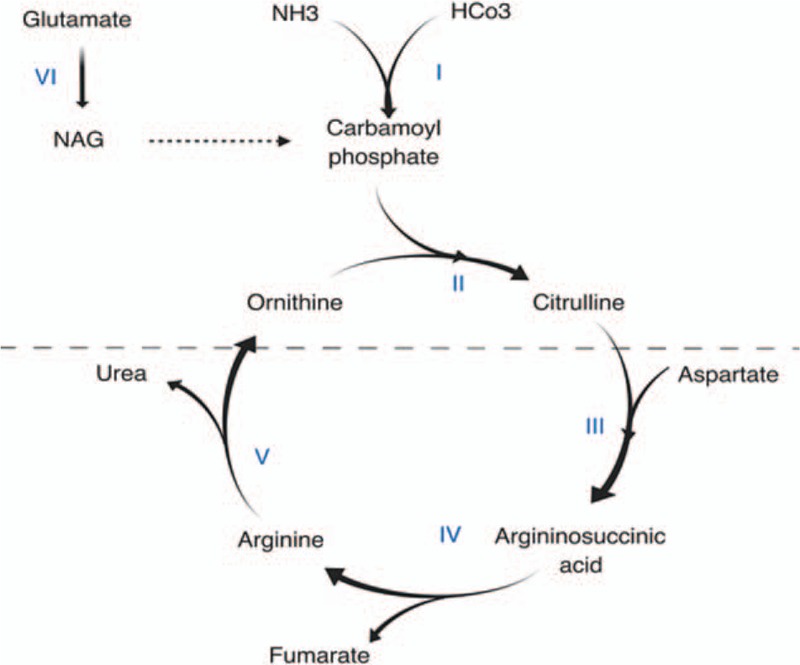
Schematic urea cycle. The enzymes involved are: I – CPS, II – OTC, III – arginosuccinate synthetase, IV – arginosuccinate lyase, V – arginase, and VI – NAG synthetase. NAG acts as an activator of CPS, which is the rate-limiting step. Reactions above the dotted line are intramitochondrial. CPS = carbamoyl phosphate synthetase, NAG = N-acetyl glutamate, OTC = ornithine transcarbamoylase.

Hyperammonemia in adults most commonly results from the impaired hepatic ability to metabolize physiologically produced ammonia. In cirrhotics, the portocaval shunting is also contributory. The role of hyperammonemia in hepatic encephalopathy associated with acute liver failure is well documented.^[13]^ Nonhepatic cases of hyperammonemia in the pediatric population usually result from inborn errors of metabolism (IEM), most commonly urea cycle disorders, organic acidurias (OA), and carnitine deficiency. The pathway of the urea cycle is depicted in Figure 3. Median age at diagnosis for OAs and urea cycle disorders is within the first year of life except for hyperornithinemia hyperammonemia homocitruliuria (HHH) and ornithine transcarbamoylase (OTC) deficiency which may not manifest till later in childhood.^[14]^ Adult-onset IEM cases presenting with hyperammonemia have been reported but are rare and are associated with other characteristic symptoms, so this discussion focuses on acquired etiologies in adult patients (Table 2).

**Table 2:**
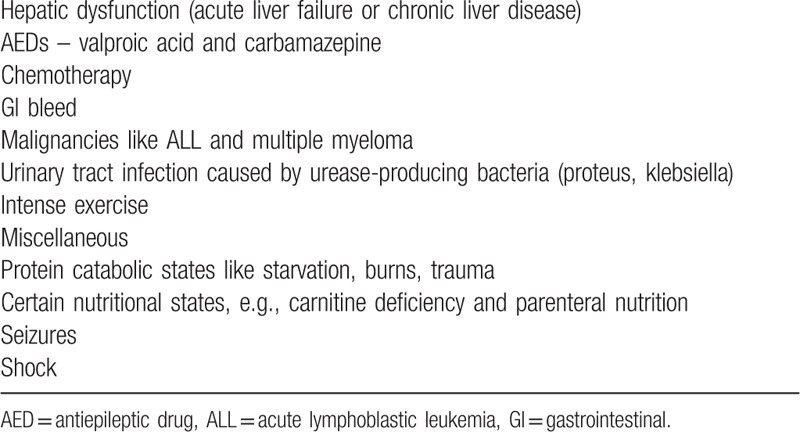
Common differential diagnoses for hyperammonemia in adults.

### 5.2 Antiepileptic drugs

Valproic acid causes dose-dependent increases in plasma ammonia levels without causing overt liver injury^[15]^ by inhibiting the activity of carbamoyl phosphate synthetase 1.^[16]^ This risk is increased with higher doses (>20 mg/kg/day) or concomitant use of other enzyme-inducing antiepileptic drugs such as phenytoin, phenobarbiturate, and carbamazepine.^[17]^ Concomitant use of topiramate also increases the risk of hepatic encephalopathy by 2 mechanisms: By blocking carbonic anhydrase and producing a degree of metabolic acidosis, which shifts the ammonium ion equilibrium towards ammonia; and By inhibiting cerebral GS preventing the conversion of ammonia and glutamate to glutamine. Interestingly, phenobarbiturate and carbamazepine also inhibit cerebral GS. Also, there are reports of hyperammonemia associated with carbamazepine monotherapy.^[18]^

### 5.3 Chemotherapy

Asparagine is a critical component for leukemic cell growth, as they lack asparagine synthetase. Asparaginase hydrolyzes asparagine, which is an otherwise nonessential amino acid, into aspartic acid and ammonia, thus directly increasing serum ammonia levels.^[19]^ Another chemotherapeutic agent associated with hyperammonemia is 5-fluorouracil (5-FU) that can cause hyperammonemia by increasing ammonia production. Administration of 5-FU induces intracellular accumulation of fluoroacetate, which is structurally similar to acetate. The fluoroacetate combines with coenzyme A to generate fluoroacetyl CoA, which replaces acetyl CoA in Kreb cycle, eventually producing fluorocitrate, which binds tightly to aconitase, thereby disrupting the cycle. Hence, transient hyperammonemia develops due to impairment of ATP-dependent urea cycle.^[20]^ As it is renally excreted, this potential is augmented with concurrent renal dysfunction. The risk is also increased during systemic infections due to increased catabolism.^[21]^

### 5.4 Gastrointestinal bleeding

**Figure 4:**
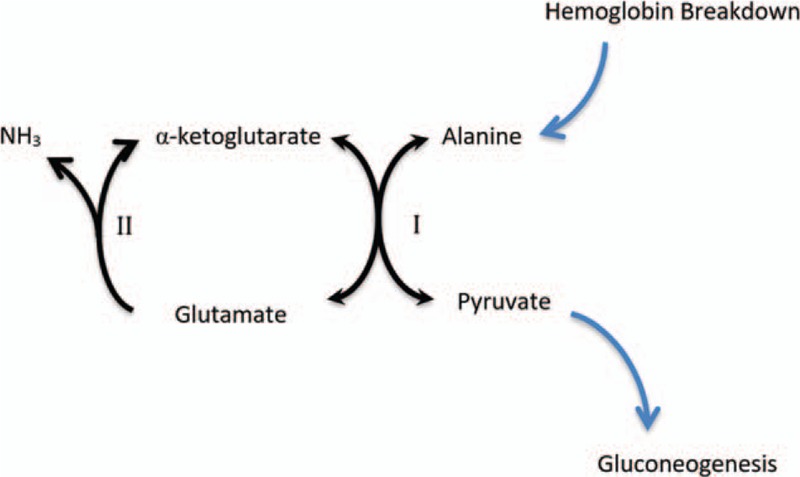
Renal tubular metabolism of alanine following GI bleed. In cirrhosis, tubular (along with remaining hepatic) ALT is upregulated secondary to hyperglucagonemia, shunting available alanine to pyruvate to promote gluconeogenesis and subsequent ammoniagenesis. The enzymes involved are: I – ALT, II – glutamate dehydrogenase. ALT = alanine aminotransferase, GI = gastrointestinal.

GI bleed had been hypothesized to cause hyperammonemia from increased protein degradation by colonic flora and mucosal oxidation, but there is also evidence of increased renal ammoniagenesis in response to elevated circulating levels of amino acids particularly glutamine and alanine.^[22]^ Patients with decompensated cirrhosis have reduced hepatic glycogen stores secondary to loss of hepatocytes and reduced enzyme activity. Reduced hepatic glycogen content produces a state of relative hyperglucagonemia.^[23]^ This subsequently promotes peripheral gluconeogenesis in renal tubules too.^[24]^ Alanine is the predominant amino acid in hemoglobin molecule^[25]^ and enters the systemic vascular pool following GI bleed. Being a gluconeogenic amino acid, it undergoes transamination in renal tubules to produce glutamate and pyruvate. Glutamate subsequently undergoes deamination to produce alpha ketoglutarate which is again available for upstream transamination, while pyruvate is used for gluconeogenesis.^[26]^ The pathway is shown in Figure 4. This cascade of reactions results in the production of ammonia, some of which is excreted in the urine and the rest is absorbed into systemic circulation.

### 5.5 Malignancies

Hyperammonemia may be seen in acute lymphoblastic leukemia, likely as a result of increased catabolism and impaired ureagenesis.^[27]^ Multiple myeloma cells have been shown to produce ammonia in vivo.^[28]^ The mechanism may involve excess protein metabolism and cytokine and immunoglobulin production.

### 5.6 Urinary tract infection (UTI)

Hyperammonemia may seldom be seen in association with UTIs with urease-producing bacteria, including proteus, klebsiella, corynebacterium, and ureaplasma.^[29]^ The urease splits urea to produce ammonia, alkalinizing the urine. With the progressive increase in pH, the ammonia equilibrium shifts from ammonium ion toward gaseous ammonia, which diffuses into circulation through inflamed urothelium.

Urea + H_2_O → 2NH_3_ + CO_2_

### 5.7 Exercise-induced hyperammonemia

Ammonia produced during intense exercise has the potential of producing central fatigue which may present with symptoms such as lethargy, incoherence, and eventually loss of consciousness.^[30]^ This condition is usually averted as ammonia is rapidly cleared from venous circulation by a normally functioning liver. However, this could become clinically significant in the case of coexistent hepatic dysfunction. Our case highlights the importance of recognition of acute onset nonhepatic hyperammonemia in the ED or inpatient setting. Regardless of the etiology, prompt treatment must be initiated. This can be achieved by decreasing ammoniagenic substrates in the colon by absorption through lactulose, or inhibition of ammonia generation through antibiotics like rifaximin or metabolic ammonia removal through sodium benzoate or ornithine-aspartate to prevent and/or treat hepatic encephalopathy. More recently, polyethylene glycol (PEG) has also been shown to be effective in reducing ammonia levels through direct laxative effect and thereby removing all nitrogenous load and potential ammoniagenic substrates from the bowel.^[31]^

### 5.8 Miscellaneous

Protein catabolic states such as trauma, burns, starvation, and steroid administration may also cause elevation of systemic ammonia levels by increasing the nitrogen load. The shock causes hyperammonemia by direct hepatic injury and increasing protein catabolism.

Total parenteral nutrition (TPN) administration may elevate serum ammonia levels by exacerbating carnitine deficiency, particularly in cirrhotics who may already be carnitine deficient.^[32]^ Carnitine is an essential factor in long-chain fatty acid metabolism, as it is involved in translocation of fatty acid residues from cytosol to mitochondria. Its deficiency causes accumulation of unoxidized fatty acids in the cytosol, which inhibit the urea cycle, thereby impairing the primary pathway of ammonia clearance.

### 5.9 Interesting facts about our case

(1) Severe lactic acidosis in our patient was also likely secondary to intense muscle activity. There is prior evidence of proportionate lactic acidosis with hyperammonemia associated with exercise.^[33]^
(2) The choice of ketamine was likely influenced by its safety^[34]^ and its potential role in the treatment of suicidal ideation in subanesthetic doses.^[35]^
(3) There have been reports of late presentation of IEM, but even with diagnosis at a late age, there is consistently a prior history of neuropsychiatric problems usually associated with digestive issues.^[36,37]^ The classical history of late-onset urea cycle defects is intermittent episodes of encephalopathy, loss of consciousness, or seizures precipitated by catabolic stress, for example, infections, surgery, pregnancy, or even a high dietary protein load. There may also be clinical evidence of neurological sequelae ranging from minimal psychomotor delay to severe mental retardation.^[38]^ Our patient did not have any such previous history or of any neurogastric attacks nor any relevant family history to suggest an IEM. Her serum amino acid profile was unremarkable, and she also seemed to have age appropriate IQ.
(4) Also, our patient did not display the typical signs and symptoms of hepatic encephalopathy likely due to the hyperacute onset of hyperammonemia and rapid resolution.

## 6 Conclusion

(1) Adult nonhepatic hyperammonemia is a relatively less common, albeit significant clinical scenario, which requires a good understanding of pathophysiology for accurate diagnosis.
(2) Differential diagnoses are broad but can usually be narrowed based on clinical presentation. Informed investigation guided by meticulous history and physical exam remain the cornerstone for early diagnosis.
(3) Regardless of the etiology, prompt treatment must be initiated to prevent hepatic encephalopathy.

## Author contributions

**Conceptualization:** Vikas Taneja.

**Formal analysis:** Vikas Taneja, Haneesh Jasuja.

**Investigation:** Vikas Taneja.

**Software:** Haneesh Jasuja.

**Writing – original draft:** Vikas Taneja, Haneesh Jasuja.

**Writing – review & editing:** Vikas Taneja.

vikas taneja orcid: 0000-0003-3638-5355.
